# Human-Specific Genes, Cortical Progenitor Cells, and Microcephaly

**DOI:** 10.3390/cells10051209

**Published:** 2021-05-15

**Authors:** Michael Heide, Wieland B. Huttner

**Affiliations:** Max Planck Institute of Molecular Cell Biology and Genetics (MPI-CBG), Pfotenhauerstr. 108, D-01307 Dresden, Germany

**Keywords:** human-specific genes, microcephaly, neocortex development, ARHGAP11B, NOTCH2NL, brain organoids

## Abstract

Over the past few years, human-specific genes have received increasing attention as potential major contributors responsible for the 3-fold difference in brain size between human and chimpanzee. Accordingly, mutations affecting these genes may lead to a reduction in human brain size and therefore, may cause or contribute to microcephaly. In this review, we will concentrate, within the brain, on the cerebral cortex, the seat of our higher cognitive abilities, and focus on the human-specific gene *ARHGAP11B* and on the gene family comprising the three human-specific genes *NOTCH2NLA*, *-B*, and *-C*. These genes are thought to have significantly contributed to the expansion of the cerebral cortex during human evolution. We will summarize the evolution of these genes, as well as their expression and functional role during human cortical development, and discuss their potential relevance for microcephaly. Furthermore, we will give an overview of other human-specific genes that are expressed during fetal human cortical development. We will discuss the potential involvement of these genes in microcephaly and how these genes could be studied functionally to identify a possible role in microcephaly.

## 1. Introduction

During human evolution, a marked increase in the size of the brain, notably of the cerebral cortex, occurred, which constitutes one essential basis for our higher cognitive abilities [1,2]. This increase in the size of the human cerebral cortex is a direct consequence of the activity and behavior of neural stem and progenitor cells (NPCs) during development [3,4,5]. Accordingly, altered NPC activity and behavior can lead to abnormal human brain size, manifested, for example, in microcephaly [6,7,8]. In the developing cerebral cortex, NPCs reside in the germinal zones of the cortical wall. In principle, two germinal zones can be distinguished: the ventricular zone (VZ), which is directly lining the ventricular surface and which mainly contains the cell bodies of apical progenitors (APs), and the subventricular zone (SVZ), which is located directly basal to the VZ and which mainly contains the cell bodies of basal progenitors (BPs) [3,4]. Furthermore, in primates and notably in humans, the SVZ is significantly expanded, and an inner (iSVZ) and an outer (oSVZ) SVZ can be morphologically distinguished [9], with the SVZ expansion pertaining primarily to the oSVZ. In the SVZ, two types of BPs can be distinguished by their morphology and their proliferative capacity. These are the basal intermediate progenitors (bIPs), which, at least in mouse, possess only limited proliferative capacity [10,11,12], and the basal radial glia (bRG), which possess a high proliferative capacity and can undergo several rounds of self-amplification [13,14,15,16]. Therefore, the oSVZ and bRG are thought to be key determinants for developing a large and highly folded cerebral cortex.

During hominid evolution, alterations in the activity and behavior of NPCs occurred, which likely underlie the large and highly folded brain that humans possess. These alterations were mainly caused by genomic changes. These changes can range from small mutations, like single nucleotide substitutions, to large changes, like the generation of novel genes [17,18,19]. Recently, genes that evolved specifically in the human lineage after the split from the lineage leading to the chimpanzee and bonobo around 7 million years ago (mya) [20,21] have come into focus, and are referred to as human-specific genes. These genes are potential candidates to exert a role in establishing the size of the human cerebral cortex as 3-fold greater than the chimpanzee cerebral cortex. Functional studies of these human-specific genes have revealed that some of them can expand the relevant NPC populations and can even increase neuronal output [22,23,24,25,26,27], indicating that human-specific genes could indeed be important players in human cerebral cortex expansion. In turn, these genes could potentially be important targets in cortical malformations. Specifically, mutations of these human-specific genes could result in or could contribute to microcephaly. We shall use the phrase “contribute to microcephaly” to refer, in general, to microcephaly that involves deletion or mutation of a gene under study.

Microcephaly is usually defined as an occipitofrontal head circumference that is more than three standard deviations below the mean for a given age, gender, and gestation [28,29,30]. In general, two types of microcephaly can be distinguished, (i) primary (congenital) microcephaly reflects defects that occurred during fetal development, and (ii) acquired (postnatal) microcephaly reflects defects that manifest during childhood [28,31,32,33,34]. Primary microcephaly can be caused by genetic factors (e.g., mutations) or external factors (like toxins, virus infections, or radiation). These factors affect the activity and behavior of NPCs, leading to a smaller NPC population or a reduced proliferative rate of NPCs, and ultimately resulting in a smaller brain [7,28,35,36]. Genes that are expressed in these NPCs and that control their activity and behavior are potentially important targets for these factors and could lead to a better understanding of primary microcephaly. In this review, we discuss human-specific genes preferentially expressed in NPCs of the developing neocortex that can promote NPC proliferation, with a focus on *ARHGAP11B* and *NOTCH2NL*. We also discuss the potential involvement of the deletion or mutation of these genes in primary microcephaly (for the sake of simplicity from now on, referred to merely as “microcephaly”).

## 2. ARHGAP11B

### 2.1. Evolution of ARHGAP11B

*ARHGAP11B* arose by a partial duplication of *ARHGAP11A* around 5 mya [37,38,39,40]. *ARHGAP11A* is a ubiquitous gene that is composed of 12 exons and encodes a RhoGAP (Rho GTPase Activating Protein) domain-containing protein [41,42]. The partial duplication of *ARHGAP11A* resulting in *ARHGAP11B* encompassed only the first 8 exons of *ARHGAP11A* [43]. This truncated copy of *ARHGAP11A* (referred to as ancestral *ARHGAP11B*) most likely did not contribute to cortical expansion. Indeed, a functional analysis of ancestral *ARHGAP11B* showed that it lacked the ability to promote BP proliferation [43]. Although the *ARHGAP11B* gene is only a truncated copy of *ARHGAP11A*, it contains the full nucleotide sequence of the RhoGAP domain. However, at the protein level, ARHGAP11B features only a truncated RhoGAP domain, lacking the last (C-terminal) 26 amino acids of this domain [43]. In line with this finding, functional analyses showed that ARHGAP11B lacks RhoGAP activity in vivo [22,43]. The truncation of the RhoGAP domain, despite the presence of the full nucleotide sequence for this domain in the *ARHGAP11B* gene, is due to a single C → G nucleotide substitution in the *ARHGAP11B* gene, which most likely occurred after the partial gene duplication event. This substitution generated a novel splice donor site in *ARHGAP11B*, leading to the loss of 55 nucleotides upon *ARHGAP11B* mRNA splicing. This loss led to a frameshift that resulted in a novel, human-specific 47 amino acid-long C-terminal sequence of ARHGAP11B [43]. In contrast to the ancestral *ARHGAP11B* gene that lacks the C → G nucleotide substitution and that would encode an ARHGAP11B protein containing a complete RhoGAP domain and exhibiting RhoGAP activity in vivo, the *ARHGAP11B* gene carrying this substitution, and the truncated RhoGAP domain-containing protein generated from it that can promote BP proliferation (see below), have often been referred to as modern *ARHGAP11B*/ARHGAP11B [43]. For the sake of simplicity, the latter will from now on be referred to merely as “*ARHGAP11B*/ARHGAP11B”.

### 2.2. Expression of ARHGAP11B during Corticogenesis

During fetal human neocortex development, *ARHGAP11B* is expressed in the VZ and SVZ (including iSVZ and oSVZ), but not in the cortical plate (CP), as shown by in situ hybridization of 13 weeks post-conception (wpc) fetal human neocortex sections [23] and by RNA-seq of micro-dissected germinal zones and CPs of fetal human neocortex ranging from 13 to 16 wpc [44]. Furthermore, RNA-seq data of FACS-isolated cell populations from fetal human neocortex enriched in either apical radial glia (aRG), bRG, or neurons (the latter also containing bRG in G1) showed that *ARHGAP11B* is expressed at almost equal levels in aRG and bRG, but not in neurons [22]. Notably, in contrast to *ARHGAP11A*, *ARHGAP11B* is not expressed in the basal end-feet of radial glia, as shown by in situ hybridization [23,45]. Furthermore, re-analysis of the Florio et al. 2015 RNA-seq data showed that one *ARHGAP11B* splice variant is specifically expressed in bRG [23].

### 2.3. Function/Role of ARHGAP11B during Corticogenesis

Functional studies of *ARHGAP11B* in different model systems revealed a potentially important role of this human-specific gene in neocortical development and evolution (summarized in Table 1). Transient overexpression of *ARHGAP11B* in mouse embryos by in utero electroporation led to an increase in BP abundance and proliferation, which was accompanied by an increase in SVZ thickness, and in half of the cases, resulted in folding of the normally unfolded mouse neocortex [22]. Furthermore, transient overexpression of *ARHGAP11B* in ferret embryos by in utero electroporation resulted in an increase in proliferative bRG, in an extension of the neurogenic period and an increase in upper-layer neurons [26]. Finally, and in contrast to the transient overexpression studies, a recent study with transgenic marmoset fetuses that expressed *ARHGAP11B* under the control of its own human promoter—hence, achieving a physiological-like expression—showed an increased size and the induction of folding of the fetal marmoset neocortex, which at the fetal stage studied, is known to be unfolded [27]. Along with this increase in size and folding, this *ARHGAP11B* expression resulted in an increase in the thickness of the CP and a specific increase in upper-layer neurons. Moreover, the oSVZ—the germinal zone thought to predominantly contribute to human neocortex expansion—was increased in thickness, which reflected an increase in the abundance of BPs, including that of the—for human neocortex expansion, highly relevant—bRG [27]. In summary, gain-of-function experiments with *ARHGAP11B* strongly suggest a key role for this gene in human neocortex expansion.

To corroborate the potentially important role of *ARHGAP11B* in fetal human neocortex development, loss-of-function experiments are needed. However, due to the high sequence identity between *ARHGAP11B* and its paralog, *ARHGAP11A* [43], and the expression of these two genes largely in the same cell types [22,23], it is difficult to find guide RNAs for specific and efficient CRISPR/Cas9-mediated disruption of *ARHGAP11B* expression, or small hairpin RNAs for specific *ARHGAP11B* RNAi. To overcome this limitation, it was recently shown that a truncated version of ARHGAP11A that comprises only its N-terminal 220 amino acids (referred to as ARHGAP11A220) can act in a dominant-negative manner on ARHGAP11B’s function [46]. Indeed, inhibition of ARHGAP11B’s function in fetal human neocortex tissue by ex vivo electroporation using *ARHGAP11A220* was found to result in a decreased abundance of BPs [46], providing the first loss-of-function evidence for an important role of *ARHGAP11B* in fetal human neocortex development.

The same study unraveled the molecular function of ARHGAP11B. In contrast to ARHGAP11A, which is mainly localized in the nucleus, ARHGAP11B is localized in mitochondria due to a functional N-terminal mitochondrial import sequence [46]. Although this mitochondrial import sequence is also present in ARHGAP11A, it appears to be functionally masked in this longer protein. Moreover, ARHGAP11A contains two nuclear localization signals that are not present anymore in the shorter ARHGAP11B protein [46]. In mitochondria, ARHGAP11B interacts with the adenine nucleotide translocase (ANT) and inhibits the opening of the mitochondrial permeability transition pore. As a result, intramitochondrial calcium levels rise, and the metabolic pathway, called glutaminolysis, a hallmark of cancer metabolism [47], is increasingly used [46]. In fact, the ARHGAP11B-induced increase in glutaminolysis was shown to be a prerequisite for the increased BP proliferation upon *ARHGAP11B* expression [46]. Interestingly, the protein encoded by a known microcephaly gene, *MCPH1*, was also shown to be associated with mitochondria and to regulate glutaminolysis [48]. Taken together, these data are consistent with the notion that an increase in glutaminolysis is associated with neocortex expansion, whereas a decrease can be a potential cause of microcephaly [49]. In summary, all the findings discussed in this section point to an important role of *ARHGAP11B* in human neocortex development and most likely in determining its size.

### 2.4. Potential Contribution of ARHGAP11B to Microcephaly

*ARHGAP11B* is located on chromosome 15, near the region that is associated with the 15q13.3 microdeletion/microduplication syndrome. The 15q13.3 microdeletion syndrome is characterized by intellectual and developmental disabilities, mostly delays in speech acquisition and in cognitive function linked to cognitive impairments [50,51,52,53]. Moreover, epilepsy/seizures are present in 28% of affected individuals [53], and the occurrence of schizophrenia was found to be increased in cohorts of 15q13.3 microdeletion patients [53,54]. In some cases, affected patients also display reduced brain volumes and microcephaly [55,56,57]. The 15q13.3 microdeletion is typically characterized by a common 2 Mb deletion on chromosome 15, including the genes *MTMR15*, *TRPM1*, *MTMR10*, *KLF13*, *OTUD7A*, *CHRNA7,* and also *ARHGAP11B* [53]. Some studies have reported homozygous deletion of this chromosomal region [55,57,58,59,60]. However, a closer examination of the coordinates of the deletions suggested that only one copy of *ARHGAP11B* is included in the deletion, or that *ARHGAP11B* is only very close to the deletion, or that the sequence of *ARHGAP11B’s* main isoform is still intact (Table 2).

Therefore, *ARHGAP11B*’s contribution to 15q13.3 microdeletion/microduplication syndrome as well as to microcephaly, in general, remains open. Due to *ARHGAP11B*’s likely role in human neocortex development and evolution (Table 1), it is conceivable that its mutation or deletion could contribute to microcephaly. Although *ARHGAP11B* has not been the focus of microcephaly studies in the past, it should be noted that, due to its high sequence homology to *ARHGAP11A*, mutations in *ARHGAP11B* in microcephaly patients were perhaps masked by *ARHGAP11A*. As some cases of 15q13.3 microdeletion display microcephaly and *ARHGAP11B* is close to one end of the deletion, it would be important to (re-)analyze 15q13.3 microdeletion cases with microcephaly in detail to see if *ARHGAP11B* is perhaps affected by the deletion.

## 3. NOTCH2NL Genes

### 3.1. Evolution of NOTCH2NL Genes

The *NOTCH2NL* gene family comprises three human-specific genes, *NOTCH2NLA*, *NOTCH2NLB,* and *NOTCH2NLC*. These genes evolved from *NOTCH2NLR*, which arose by gene duplication from *NOTCH2* and exist as pseudogenes in chimpanzee and gorilla. During human evolution, a gene conversion occurred between *NOTCH2NLR* and *NOTCH2*, which restored *NOTCH2NLR’s* function [25,61]. Sequential duplications then generated *NOTCH2NLB*, followed by *NOTCH2NLA*, and finally, *NOTCH2NLC* [61]. The genomic sequences of the four *NOTCH2NL* paralogs include the *NOTCH2* promoter and six N-terminal epidermal growth factor (EGF)-like domains from *NOTCH2* exons 1 to 4 but exclude the transmembrane and cytoplasmic domains of NOTCH2 [24,25]. This protein sequence corresponds to the NOTCH2 protein encoded by the shortest *NOTCH2* splice variant [23]. In addition, the *NOTCH2NL* genes contain a fifth exon that is unique to these genes and corresponds to an intronic region of *NOTCH2*, resulting in 20 amino acids that are unique to NOTCH2NL proteins [24,25]. NOTCH2 normally contains a signal peptide at the N-terminus, directing it to the secretory pathway. However, of the human-specific *NOTCH2NL* genes, only *NOTCH2NLB* encodes a signal peptide, suggesting that only NOTCH2NLB would be secreted via the canonical secretory pathway [24,25]. Interestingly, the human-specific *NOTCH2NL* genes co-evolved as pairs with other human-specific genes [61]. These genes belong to the *NBPF* (Neuroblastoma breakpoint family) gene family. Specifically, *NOTCH2NLA* co-evolved with *NBPF10*, *NOTCH2NLB* co-evolved with *NBPF14*, and *NOTCH2NLC* co-evolved with *NBPF19* [61].

### 3.2. Expression of NOTCH2NL Genes during Corticogenesis

In situ hybridization data showed that the *NOTCH2NL* genes are expressed in the germinal zones of fetal human neocortex, in most, if not all, cells of the VZ and in a few scattered cells of the iSVZ and oSVZ, indicating that these genes are expressed mainly in APs and in some BPs [23,24]. Consistent with this, a re-analysis of RNA-Seq data of cell populations isolated from fetal human neocortex [22] using the Kallisto algorithm showed that *NOTCH2NL* genes are indeed expressed mainly in APs [23]. The single-cell RNA-seq datasets analyzed by Suzuki et al. 2018 and Fiddes et al. 2019 showed that *NOTCH2NL* expression closely resembles that of *NOTCH2* [24,61]. Additionally, *NOTCH2NL* genes show very similar expression patterns in the developing human cortex in comparison to another human-specific gene, *NBPF10*, with high expression in radial glial cells [61]. Moreover, RNA-seq analysis of fetal human cortex ranging from 7 to 21 gestational weeks showed that *NOTCH2NLB* exhibits the strongest expression of the *NOTCH2NL* paralogs, followed by *NOTCH2NLA*, and, at significantly lower expression levels, *NOTCH2NLC* [24].

### 3.3. Function/Role of NOTCH2NL Genes during Corticogenesis

Three studies originally described the function of the human-specific *NOTCH2NL* genes during neocortex development (summarized in Table 1). Florio, Heide, and colleagues [23] analyzed the role of *NOTCH2NLA*. Overexpression of this gene in embryonic mouse neocortex by in utero electroporation resulted in an increase in cycling BPs in the SVZ and intermediate zone, but not in cycling APs in the VZ [23]. Moreover, this increase involved *Tbr2*-expressing but not *Sox2*-expressing BPs, indicating that bIPs rather than bRG were affected. Furthermore, a decrease in BP cell cycle exit was observed [23].

Vanderhaeghen and colleagues [24] analyzed the role of *NOTCH2NLB*. in utero electroporation of *NOTCH2NLB* in embryonic mouse neocortex resulted in an increased number of Pax6-positive cells in the VZ, indicating that *NOTCH2NLB* can maintain cells in the VZ and in the progenitor state [24]. In contrast to the findings with *NOTCH2NLA* [23], no effects on BPs were observed upon *NOTCH2NLB* overexpression. Moreover, lentiviral overexpression of *NOTCH2NLB* in cortical progenitors differentiated from human embryonic stem cells resulted in a larger clone size and an increase in neuronal output [24]. This increase in clone size by *NOTCH2NLB* overexpression was found to be mediated by a decrease in cell cycle exit and an increase in cell cycle re-entry [24].

Jacobs, Haussler, and colleagues [25] ectopically expressed *NOTCH2NL* isoforms that corresponded to *NOTCH2NLA* and *NOTCH2NLB* in mouse cortical organoids, but did not find obvious differences in comparison to control organoids [25]. However, RNA-seq analysis of day-6 organoids showed an increased expression of genes that are associated with negative regulation of neuronal differentiation. Furthermore, deleting *NOTCH2NL* genes using the CRISPR/Cas9 technology (producing a clone with a homozygous deletion of both *NOTCH2NLA* and *NOTCH2NLB* and a heterozygous deletion of *NOTCH2NLC*) resulted in smaller human cortical organoids with increased Ctip2 protein levels [25]. Moreover, transcriptome analysis of these *NOTCH2NL* KO organoids revealed an upregulation of genes involved in neuronal differentiation [25]. In summary, these three studies [23,24,25] indicate a role of the human-specific *NOTCH2NL* genes in keeping NPCs in the developing neocortex in a proliferative state.

As to the molecular function of *NOTCH2NL* genes, it was shown that the NOTCH2NL proteins can physically interact with NOTCH2 and can enhance not only NOTCH2 activation but also the activation of NOTCH1 and NOTCH3, indicating that NOTCH2NL proteins can, in general, enhance Notch receptor activation [25]. Specifically, for *NOTCH2NLB*, it was shown that its overexpression leads to the upregulation of *Hes1*, a direct downstream effector of the Notch pathway [24]. Moreover, *NOTCH2NLB* mutants with deletions of specific protein domains revealed that the EGF repeats of *NOTCH2NLB* are critical for its function, in contrast to the C-terminal domain. Specifically, the EGF repeats of NOTCH2NLB were found to be required for a direct interaction with the Notch ligand Delta-like 1 (DLL1) [24], which is important for the regulation of progenitor proliferation vs. differentiation [62]. This interaction (when the EGF repeats are present) leads to a reduction of functionally available DLL1 protein at the cell surface in *NOTCH2NLB*-expressing cells and to cortical NPC expansion [24].

### 3.4. Potential Contribution of NOTCH2NL Genes to Microcephaly

The three human-specific *NOTCH2NL* genes, together with the three *NBPF* genes with which they co-evolved, are localized on chromosome 1, close to the region that is associated with the 1q21.1 distal syndrome [24,25]. Depending on whether this region is duplicated or deleted, this syndrome is associated with either macrocephaly or microcephaly, respectively [63,64]. The first studies describing the 1q21.1 distal syndrome used the GRCh37 human genome assembly for their analyses, in which *NOTCH2NL* genes were incorrectly positioned and not included in the duplicated or deleted region. Therefore, Jacobs, Haussler, and colleagues [25] re-mapped previous CNV microarray data of 11 1q21.1 distal syndrome patients to the GRCh38 assembly and found that *NOTCH2NLA* and *NOTCH2NLB* were located within the region associated with this syndrome. Moreover, all nine microcephaly cases re-analyzed in this study showed a *NOTCH2NLA* and/or *NOTCH2NLB* deletion [25]. These data indicate that the loss of *NOTCH2NLA* and *NOTCH2NLB* could be an important contributor to microcephaly. Moreover, as two other human-specific genes are directly adjacent to *NOTCH2NLA* and *NOTCH2NLB—*that is, *NBPF10* and *NBPF14*, respectively—mutations affecting these genes could be potentially important contributors to microcephaly. These considerations lead us to a discussion of other human-specific genes and their potential roles in microcephaly.

## 4. Other Human-Specific Genes Expressed during Fetal Human Brain Development

### 4.1. Identification of Human-Specific Genes with a Potential Role in Brain Development

As described above, *ARHGAP11B* and *NOTCH2NL* genes exert important roles during fetal human neocortex development and most likely also contribute to microcephaly. However, as these are not the only human-specific genes preferentially expressed in cortical NPCs, other human-specific genes could, in addition, be involved in microcephaly. One prerequisite is that such genes are expressed during fetal human neocortex development and preferably in NPCs. In this context, we wish to discuss three independent studies that used different approaches to identify human-specific genes that are expressed during cortical development.

First, Florio, Heide, and colleagues [23] re-analyzed five published, methodologically different transcriptome datasets of fetal human neocortex [22,44,65,66,67] for protein-coding genes that are either preferentially expressed in the germinal zones (VZ and SVZ) in comparison to the remaining zones of the developing neocortical wall (e.g., CP) or preferentially expressed in cortical NPCs in comparison to neurons. These genes, referred to as cortical NPC-enriched, were then analyzed for their occurrence in primates vs. non-primate mammals and their appearance in the various primate clades. This led to the identification of 50 primate-specific genes, of which 15 are human-specific protein-coding genes that are preferentially expressed in cortical NPCs (Table 3); these 15 genes include *ARHGAP11B* and *NOTCH2NLA* [23].

Second, Vanderhaeghen and colleagues [24] generated RNA-seq data from fetal human neocortex ranging from 7 to 21 gestational weeks. They focused their analysis on hominid-specific duplicated gene families (containing genes duplicated in the hominid and human lineage). In this study, an expression level threshold of 5 fragments per kilobase million (FPKM) was defined, as this was the minimal expression level of a set of 60 known marker genes of corticogenesis [24]. Then, hominid-specific duplicated gene families were considered of relevance when at least two paralogs were expressed above this threshold. With this approach, the authors identified 24 gene families, comprising 68 genes, that fulfilled these criteria. Notably, half of these genes were preferentially expressed in NPCs or at early stages of corticogenesis. Of these 68 genes, 35 are human-specific and display an intact open reading frame (Table 3) [24]. These 35 human-specific genes include *NOTCH2NLB*; however, they do not include *ARHGAP11B*, as in this dataset, *ARHGAP11B*’s expression was below the 5 FPKM threshold [24].

Third, Eichler and colleagues [68] used an approach that allowed them to specifically study the expression of human-specific genes without the contribution of the respective ancestral genes. To accomplish this, they used reverse transcriptase (RT) template switching to enrich for full-length cDNA molecules [68]. Next, they enriched cDNAs originating from paralogous loci by using a complementary oligonucleotide capture panel. Finally, PacBio sequencing technology was used to generate sufficiently long reads that would allow for distinguishing between paralogs [68]. This study used RNA from developing and adult whole brain pooled from multiple individuals. Out of the original set of 39 duplicated gene families screened, they focused on 19 gene families for a more detailed analysis, comprising 31 human-specific genes. Of these 31 genes, 29 exhibit expression in the brain (Table 3) [68]. This dataset includes *NOTCH2NLA* and -*B,* and *ARHGAP11B*.

### 4.2. Overview of Other Human-Specific Genes That Could Contribute to Microcephaly

The human-specific genes identified in the above-described three independent studies [23,24,68] can only have a role in fetal human brain development and in microcephaly if they are expressed in the fetal human brain. As the above-described datasets used different approaches to identify human-specific genes with such an expression, genes to be considered as candidates to have a role in fetal human brain development should be shared between at least two of the three datasets. This is the case for 11 of the human-specific genes (Table 3). These are *ARHGAP11B*, *NOTCH2NLA*, and *NOTCH2NLB*, already described above, and *FAM72B*, *FAM72C*, *FAM72D*, *GTF2IP1*, *GTF2IRD2B*, *NBPF14*, *SRGAP2B*, and *SRGAP2C*, which are described in the following.

FAM72B (Family With Sequence Similarity 72 Member B), FAM72C (Family With Sequence Similarity 72 Member C), and FAM72D (Family With Sequence Similarity 72 Member D) evolved most likely by three duplication events of FAM72A (Family With Sequence Similarity 72 Member A) in conjunction with the duplication of the SRGAP2 genes [69]. A functional study of the ancestral gene FAM72A showed that the knock-down of this gene in primary adult NPCs by small hairpin RNAs leads to reduced proliferation of NPCs and their increased differentiation to the neuronal lineage, indicating an important role for FAM72A in the balance between proliferation and differentiation [70]. This suggests that FAM72A could be an interesting candidate gene to potentially cause microcephaly when deleted or mutated. As the four FAM72 genes show very high homology to each other, it is likely that FAM72B-D fulfills similar roles as FAM72A in human brain development and maybe also in microcephaly.

*GTF2IP1* (*General Transcription Factor IIi Pseudogene 1*) arose by fusion of the products of a partial duplication of the ancestral gene *GTF2I* (*General Transcription Factor IIi*) and of a duplication of the promoter and the first exon of *GATSL2* (*GATS-Like Protein 2*) [68]. The ancestral gene *GTF2I* is a transcriptional regulator and is deleted in 7q11.23 Williams–Beuren syndrome [71]. *GTF2IP1* is also located in the breakpoint regions of this syndrome [72] and could potentially contribute to this disease. Williams–Beuren syndrome is characterized by a wide spectrum of symptoms that vary greatly in range and severity [73]. However, one common feature of Williams–Beuren syndrome is that affected patients have smaller brain volumes compared to healthy individuals [74,75,76,77,78], indicating that the genes affected in Williams–Beuren syndrome contribute to microcephaly.

*GTF2IRD2B* (*GTF2I Repeat Domain Containing 2B*) is a complete duplication of the ancestral gene *GTF2IRD2* (*GTF2I Repeat Domain Containing 2*) [68]. Due to the complete duplication, this gene most likely has a similar function as the ancestral gene, which acts as a TFII-I type of transcription factor [79]. Like *GTF2IP1*, this gene is also localized in the region of chromosome 7, which is affected in Williams–Beuren syndrome [80], indicating that mutations affecting this gene could also contribute to microcephaly.

*NBPF14* (*Neuroblastoma Breakpoint Family Member 14*) is an interesting candidate for a role in microcephaly, not only as it co-evolved with *NOTCH2NLB* and is located in the chromosomal region that is associated with 1q21.1 distal syndrome [24,25], but also as it belongs to the *NBPF* gene family. This family of genes most likely evolved from the gene encoding the centrosome-associated protein myomegalin [81] and appears only in placental mammals, with a large number of members present in primates and the highest number of members present in humans [82]. The *NBPF* genes contain multiple copies of the so-called Olduvai domain. This domain was previously known as DUF1220 (domain of unknown function 1220) [83]. Concomitant with the increase in the number of *NBPF* genes in the human lineage, a striking increase in the copy number of this domain was observed [84]. Moreover, a high number of copies of this domain has been found to be correlated with brain size and the severity of autism, whereas a low number of copies has been found to be correlated with the severity of schizophrenia [81,85]. Functional studies showed that overexpression of *NBPF15*, which contains multiple copies of this domain, can increase the number of neural stem cells that can be generated from human embryonic stem cells [86]. These data, together with the facts that *NBPF14* co-evolved with *NOTCH2NLB* and is located in the chromosomal region affected in 1q21.1 distal syndrome, make this gene as well as the other members of the *NBPF* gene family interesting candidates for a role in microcephaly.

*SRGAP2B* (*SLIT-ROBO Rho GTPase Activating Protein 2B*) and *SRGAP2C* (*SLIT-ROBO Rho GTPase Activating Protein 2C*) evolved by two partial duplications of the ancestral gene *SRGAP2A* and contain the majority of the F-BAR domain, but not the RhoGAP and SH3 domains, of *SRGAP2A* [38,87]. As *SRGAP2C* is more strongly expressed in the human brain than *SRGAP2B* [87], functional analyses focused mainly on *SRGAP2C*. SRGAP2C can interact with and inhibit the function of SRGAP2A, leading to an increased rate of neuronal migration, neoteny during dendritic spine maturation, increased spine density, and longer spines [88]. Furthermore, it was shown that through this inhibition of SRGAP2A function, SRGAP2C can increase excitatory and inhibitory synapse density and delay synapse maturation [89]. Moreover, it was shown that SRGAP2C is more potent in its ability to inhibit synaptic maturation and to maintain increased spine density than SRGAP2B [90]. In summary, these data do not suggest a direct involvement of these genes in microcephaly.

### 4.3. Brain Organoids as an Ideal Model System to Study the Potential Contribution of Human-Specific Genes to Microcephaly

One limitation for functional analyses of human-specific genes is the availability of suitable model systems. For gain-of-function studies, which are highly relevant with regard to the evolution of the human neocortex, several model systems are available, ranging from rodents to non-human primates. For loss-of-function studies, however, which are particularly important for microcephaly research, only very few model systems are available. One very promising model system for the latter type of studies are brain organoids [91,92]. Brain organoids are three-dimensional (3D) cell assemblies generated from embryonic or induced pluripotent stem cells that recapitulate many hallmarks of developing neocortical tissue, including the main features of the tissue architecture and the cell-type composition [93,94]. This model system has already been successfully used to study the microcephaly genes *CDK5RAP2*, *CPAP, ASPM*, *WDR26*, and *IER3IP1* [92,95,96,97,98]. One key requirement for human brain organoids to be a useful model system for such studies is that these organoids express the candidate human-specific genes of interest. Several studies have analyzed the transcriptional landscape of human brain organoids (e.g., [99,100,101,102,103]) and have provided gene expression data that can be screened for the expression of the human-specific gene of interest. Of note, Camp, Treutlein, and colleagues [103] specifically studied the expression in cerebral organoids of 24 human-specific genes that were described in the datasets of Florio, Heide, and colleagues [23] (all 15 genes), Vanderhaeghen and colleagues [24] (4 genes), and Eichler and colleagues [68] (14 genes) (see also Table 3). All of the genes examined, with the exception of *FCGR1B*, were found to be expressed in cerebral organoids [103]. This suggests that brain organoids can be used to study the function of such genes. For example, such brain organoid-expressed genes can be knocked out, and the effect of the KO on NPC activity and behavior can be studied in brain organoids. This could provide first indications for a potential role of such genes in microcephaly.

## 5. Conclusions

In conclusion, there is a high probability that mutations of human-specific genes can contribute to microcephaly. Notably, due to their role in fetal human neocortex development and their (potential) involvement in microdeletion syndromes, the genes *ARHGAP11B* and *NOTCH2NL* are likely targets for mutations leading to microcephaly. Furthermore, the other above-described human-specific genes could also contribute to microcephaly, especially the ones for which the ancestral gene is already known to be involved in microcephaly. However, (further) functional analyses of these human-specific genes are required. Specifically, loss-of-function experiments for these candidate genes are needed, as these are the most relevant experiments to identify a potential contribution to microcephaly. Brain organoids can be an ideal platform for such loss-of-function experiments. Importantly, all the knowledge in basic science obtained for these human-specific genes needs to be transferred to clinical research to get a better understanding of microcephaly. In case an involvement of any of these genes in microcephaly is uncovered, those genes should be added to the list of potential risk factors and targets for genetic screenings.

## Figures and Tables

**Table 1:** Summary of the main functions of *ARHGAP11B* and of the *NOTCH2NL* genes during neocortex development.

*ARHGAP11B*	*NOTCH2NLA*	*NOTCH2NLB*
neocortex size ↑	basal progenitors ↑	apical progenitor ↑
cortical folding ↑	basal intermediate progenitors ↑	cortical progenitor proliferation ↑
cortical plate thickness ↑		cortical progenitor differentiation ↓
upper-layer neurons ↑		neuronal output ↑
oSVZ thickness ↑		
basal progenitors ↑		
basal radial glia ↑		

**Table 2:** Genomic coordinates (GRCh37/hg19 assembly) of *ARHGAP11B* (left) and of patients reported to have a homozygous 15q13.3 microdeletion (see references indicated).

ARHGAP11B	Le Pichon et al., 2010 [58]	Endris et al., 2010 [55]	Spielmann et al., 2011 [59]	Le Pichon et al., 2013 [60]	Masurel-Paulet et al., 2014 [57]
chr15:30,918,879–30,931,023	chr15:30,931,644–32,914,281	chr15:29,085,644–32,511,004 (paternal) ^1^ chr15:31,122,986–32,511,004 (maternal) ^1^	chr15:30,971,330–32,439,084	chr15:30,931,644–32,914,281	chr15:30,938,215–32,510,863 (three patients)

^1^ Patient 2.

**Table 3:** Human-specific genes that are expressed in the developing and/or adult brain (in alphabetical order). Bold font indicates the 11 genes that were identified in two studies.

Florio et al., 2018 [23]	Suzuki et al., 2018 [24]	Dougherty et al., 2018 [68]
*ANKRD20A2*	*C15orf40*	***ARHGAP11B***
*ANKRD20A4*	*CROCCP2*	*ARHGEF34P*
***ARHGAP11B***	*FAM103A1*	*ARHGEF35*
*CBWD6*	*GOLGA8A*	*BOLA2*
*DHRS4L2*	*GOLGA8B*	*CD8B2*
*FAM182B*	*GOLGA6L9*	*CHRFAM7A*
***FAM72B***	***GTF2IP1***	***FAM72B***
***FAM72C***	***GTF2IRD2B***	***FAM72C***
***FAM72D***	*GUSBP11*	***FAM72D***
*GTF2H2C*	*LINC00957*	*FCGR1B*
*NBPF10*	*LOC100093631*	*FCGR1CP*
***NBPF14***	*LOC100288778*	*FRMPD2B*
***NOTCH2NLA***	*LRRC37A2*	*GPR89B*
*SMN2*	*LRRC37B*	***GTF2IP1***
*ZNF492*	*NBPF1*	*GTF2IP4*
	*NBPF8*	***GTF2IRD2B***
	*NBPF9*	*HYDIN2*
	***NBPF14***	*NCF1C*
	*NBPF15*	*NCF1B*
	***NOTCH2NLB***	***NOTCH2NLA*** ^1^
	*NPIPA5*	***NOTCH2NLB*** ^1^
	*NPIPB3*	*NOTCH2NLC* ^1^
	*NPIPB5*	*NOTCH2NLD* ^1^
	*PDE4DIPB*	*PTPN20*
	*PMS2CL*	*ROCK1P1*
	*POLR2J3*	*SLX1B*
	*POTEE*	***SRGAP2B***
	*POTEJ*	***SRGAP2C***
	*RASA4B*	*SRGAP2D*
	*RASA4CP*	
	***SRGAP2B***	
	***SRGAP2C***	
	*WASH2P*	
	*WASH3P*	
	*WASH7P*	

^1^ Note: fused to *NBPF* genes.
